# Life Gain in Italian Smokers Who Quit

**DOI:** 10.3390/ijerph110302395

**Published:** 2014-02-26

**Authors:** Laura Carrozzi, Franco Falcone, Giulia Carreras, Francesco Pistelli, Giuseppe Gorini, Andrea Martini, Giovanni Viegi

**Affiliations:** 1 Pulmonary Unit, CardioThoracic and Vascular Department, University Hospital of Pisa, via Paradisa 2, Cisanello, Pisa 56124, Italy; E-Mails: l.carrozzi@ao-pisa.toscana.it (L.C.); f.pistelli@ao-pisa.toscana.it (F.P.); 2 Unit of Pulmonary Environmental Epidemiology, Institute of Clinical Physiology, Italian National Research Council (IFC-CNR), via Trieste 41, Pisa 56126, Italy; E-Mail: viegig@ifc.cnr.it; 3 Italian Association of Hospital Pulmonologists (AIPO) Research, Via Antonio Da Recanate, 2, Milan 20124, Italy; E-Mail: franco.falcone@aiporicerche.it; 4 Unit of Environmental and Occupational Epidemiology, Cancer Prevention and Research Institute (ISPO), via delle Oblate 2, Florence 50139, Italy; E-Mails: g.gorini@ispo.toscana.it (G.G.); a.martini@ispo.toscana.it (A.M.); 5 Institute of Biomedicine and Molecular Immunology, Italian National Research Council (IBIM-CNR), via Ugo La Malfa 153, Palermo 90146, Italy

**Keywords:** smoking cessation, Italy, survival

## Abstract

This study aims to estimate the number of life years gained with quitting smoking in Italian smokers of both sexes, by number of cigarettes smoked per day (cig/day) and age at cessation. All-cause mortality tables by age, sex and smoking status were computed, based on Italian smoking data, and the survival curves of former and current smokers were compared. The more cig/day a man/woman smokes, and the younger his/her age of quitting smoking, the more years of life he/she gains with cessation. In fact, cessation at age 30, 40, 50, or 60 years gained, respectively, about 7, 7, 6, or 5, and 5, 5, 4, or 3 years of life, respectively, for men and women that smoked 10–19 cig/day. The gain in life years was higher for heavy smokers (9 years for >20 cig/day) and lower for light smokers (4 years for 1–9 cig/day). Consistently with prospective studies conducted worldwide, quitting smoking increases life expectancy regardless of age, gender and number of cig/day. The estimates of the number of years of life that could be gained by quitting smoking, when computed specifically for a single smoker, could be used by physicians and health professionals to promote a quit attempt.

## 1. Introduction

Smoking cessation has major and immediate health benefits for persons with and without smoking-related diseases, and smoking men and women who quit live longer than those who continue smoking [1,2,3]. The seminal prospective study by Doll and Peto [4], has shown that smokers on average die about 10 years younger than non-smokers, while smoking cessation at age 50 halves and at age 30 avoids almost all the hazard. The Million Women Study [5] has demonstrated that also the first generation of UK women born around 1940, which smoked like men, shows hazards and benefits similar to male smokers. Similar figures have been observed in the US population on data from the Cancer Prevention Study 2 (CPS2) [6], and more recently from the US National Health Interview Survey [7] and other seven US population surveys [8]. Few data on life expectancy in smokers come from European countries other than UK [9], while figures similar to those observed in the US and UK come from Japan [10].

Estimates of risks associated with smoking are useful to provide a sounder scientific basis for public health messages and clinical advice. Yet, in order to promote a quit attempt, a challenging task for health professionals is to efficiently communicate to the smoker what he/she can obtain by quitting smoking. A strong health message may be the “Life gain”: that is, the additional number of years of life that a male or female smoker can live if he/she stops smoking, at his/her own age, his/her usual number of daily cigarettes.

To the best of our knowledge, there are no studies that have evaluated survival in relation to quitting smoking in Italian smokers; moreover, existing studies do not take into account the number of cigarettes smoked per day to estimate life expectancy associated with smoking cessation. Based on Italian data, the present study quantifies gain in life expectancy associated with quitting smoking for both men and women, by age of quitting, and number of cigarettes smoked per day. 

## 2. Material and Methods

### 2.1. Data

The age and sex-specific number of deaths and population, as well as the age and sex-specific death rates for all causes were extracted from the Italian life tables for the year 2009 [11]. Age and sex-specific prevalence rates of current smokers (CS), never smokers (NS), and former smokers (FS) from the Multipurpose Survey for people aged 25 years and over carried out in 2011 by the Italian Institute of Statistic (ISTAT) were used [12]. This survey was carried out on a representative sample of the Italian population of about 24,000 families and 54,000 persons distributed in about 850 Italian municipalities.

Since ISTAT survey did not provide proportions of FS by time since smoking cessation, these figures (cessation from 1–2, 3–5, 6–10, 11–15, and >15 years) for people aged 25 years and over were obtained by another Italian survey on smoking habits for the year 2009. This survey was carried out by DOXA, the Italian branch of the Gallup International Association, on a representative sample of about 3,500 persons [13]. Relative risks (RRs) of death for CS and FS were extracted from Cancer Prevention Study 1 (CPS1) [14] and CPS2 [15].

### 2.2. Statistical Analyses

We computed life tables specific for CS, NS, FS. We first computed age and sex-specific all-cause death rates for NS [16]. We considered the population stratified in the mutually exclusive groups of CS, NS, and FS. Age and sex-specific death rates for all causes (DR) are a weighted sum of age and sex-specific death rates for CS, NS, and FS:

*DR* = *P_CS_* · *DR_CS_* + *P_NS_* · *DR_NS_* + *P_FS_* · *DR_FS_* (1)

where P_CS_, P_NS_ and P_FS_ are the age and sex-specific smoking prevalence rates respectively of CS, NS and FS in the population.

Since the relative risks of death for FS and CS (RR_FS_, RR_CS_) are defined respectively as the ratio between the corresponding death rates and the death rates for NS:

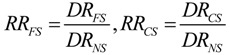

inverting Equation (1), we obtained the NS death rates:

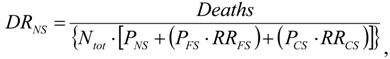

where Deaths and N are the age and sex-specific 2009 number of deaths and population, respectively.

Relative risks of death for CS by gender and smoked cig/day (1–9, 10–19, ≥20) were estimated by proportioning data from CPS1, since RRs by cig/day were not available from the more recent CPS2. CPS1 RRs were proportionated in order to generate overall (not cig/day-specific) RRs according to those from CPS2. Relative risks for men were then further proportionated in order to be in agreement with those estimated for another European country (Norway) by Bjartveit and Tverdal [17]. The latter computation was carried out only for men, since smoking epidemic of Northern European women was different with respect to Italian women [17,18]. 

Relative risks of death for FS were stratified for time since smoking cessation by modelling RRs with a negative-exponential curve starting from the value for CS and converging to the value 1 with a rate of convergence decreasing with age to account for the cumulative nature of the effects of smoking [19]. Doing so, RRs for FS are assumed similar to those of CS immediately after cessation, and become similar to those of NS, *i.e.*, they attain value 1, in the long run:

*RR_FS_* = 1 + (*RR_CS_* − 1) · exp(−*γ*(*a*) · *s*)

*γ*(*a*) = *γ*_0_ · exp(−*η* · *a*)

with a age; s time since smoking cessation; γ regression coefficient of time dependency γ_0_; η intercept and regression coefficient respectively of age dependency. Point estimates of the parameters γ_0_ and η were respectively 0.11 and 0.01, estimated using the major cohort studies presented in literature [19].

All RRs were then converted into death rates and the latter were used to compute life tables specific for gender and daily number of cigarettes. The proportions expected to survive from one age to another were calculated by multiplying together the relevant five-year age specific survival probabilities. These probabilities are calculated as exp (−5R), where exp is the exponential function and R is the annual death rate (deaths/person years) in that age range [4].

The survival curves of CS and FS were then compared to estimate the number of life years gained with quitting smoking at various ages. This was done by computing the maximum distance between the two survival curves [4].

## 3. Results and Discussion

### 3.1. Results

**Figure 1:**
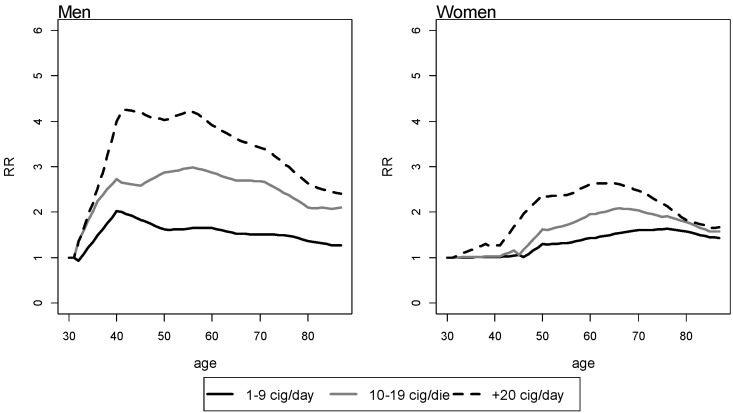
Relative risks of death for current smokers by number of cigarettes smoked per day and sex.

The trend with age of RRs of death for CS by number of cig/day is shown for men and women in Figure 1. Before age 30, the risks of death for CS and FS are equal to those for NS. In both genders, RRs of death increase with age until a peak, while declining afterward, and are higher in heavier smokers (dose-response effect). In men RRs of death peak more rapidly and at younger age (about 40 *vs.* 60 years), and are higher (about one and half) than in women.

**Figure 2:**
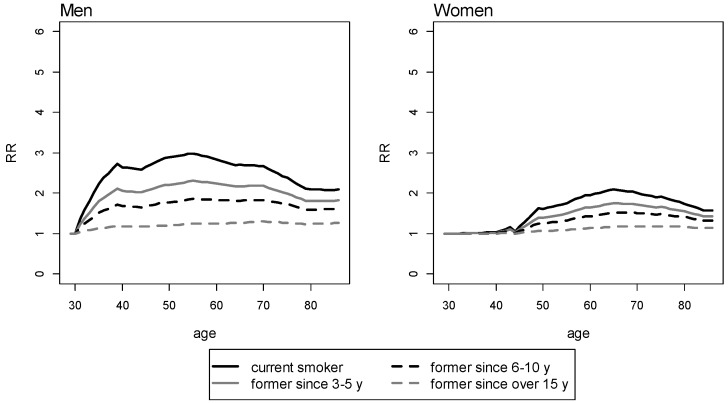
Relative risks of death for current and former smokers who smoked 10–19 cig/day by number of years since quitting smoking and sex.

Both in men and women, RRs of death decrease with increasing the number of years since smoking cessation, and the magnitude of reduction is directly proportional to the number of cig/day smoked at the time of quitting smoking. As an example, the RRs for FS who smoked 10–19 cig/day, stratified by the number of years since smoking cessation, after modelling them as negative exponential curves based on RRs for CS, are shown in Figure 2. For those FS who quit since over 15 years, the RRs of death are near to 1 throughout the age range.

**Figure 3:**
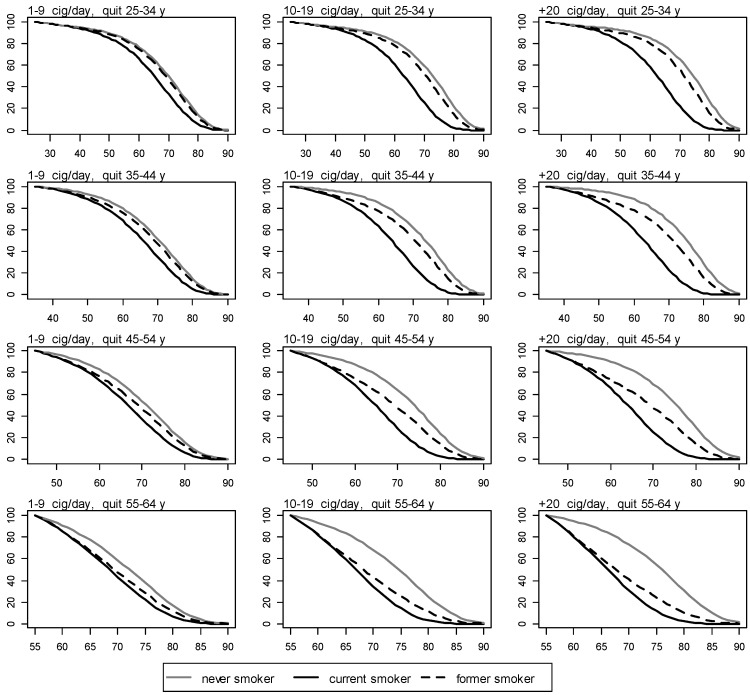
Survival for never, current, and former men smokers by number of cigarettes smoked per day and age of quitting. y axis: proportion of survival. x axis: age in years.

**Figure 4:**
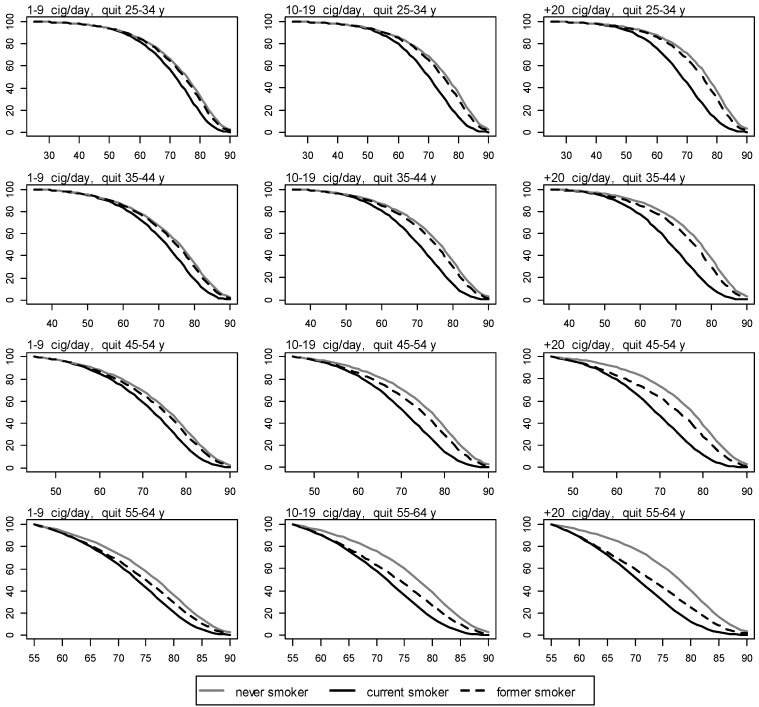
Survival for never, current, and former women smokers by number of cigarettes smoked per day and age of quitting. y axis: proportion of survival. x axis: age in years.

The survival curves for NS, CS, and FS, by cigarettes smoked per day and age of quitting, are shown in Figure 3 and Figure 4 for men and women, respectively. 

The more the cig/day a man or a woman smokes, and the younger the age of quitting smoking, the more the life extension following smoking cessation. When smoking less than 20 cig/day is stopped before 35 years of age, life expectancy resembles that of a NS for both men and women. When smoking is stopped after 35 years of age, lower proportions of survival are observed in former smoking men than in former smoking women in all groups of cigarettes smoked per day.

The number of years of life that a man or a woman could gain by quitting smoking, at a specific age class by cig/day, is reported in Table 1. A steady trend in the number of years of life gained with increasing number of cig/day and with decreasing age at cessation is present. Quitting smoking at all ages determines a meaningful gain in life expectancy, particularly for those with heavier cigarette consumption. Overall, life gain is higher in men than in women. As an example, a man smoking 1–9, 10–19, or over 20 cig/day, who quit at 35–44 years of age, may gain 3, 7, or 8 years of life, respectively. The corresponding figures for a woman are slightly lower: 3, 5, and 6 years, respectively. Even smoking cessation at 55–64 determines a gain in life expectancy, especially for those who smoked more than 20 cig/day (6 and 4 years for men and women, respectively). However, earlier stopping is associated with lager benefits. Along with the number of years gained with smoking cessation, Table 1 shows life expectancy if continuing smoking, by intervals of age at cessation and smoked cig/day. Figures show a higher percentage increase in life gain in older smokers. As an example, a man smoking more than 20 cig/day who quit at 55–64 years of age, gains a 33% of life (*i.e.*, 6 years gained with smoking cessation over 18 years of additional life expectancy if continuing smoking) as compared to a 20% of additional life (9 over 45 years, respectively) for a man smoking the same number of cig/day but quitting at 25–34 years of age.

**Table 1:** Number of years of life gained and life expectancy if continuing smoking (in parenthesis) by age at smoking cessation, number of cigarettes smoked per day and gender.

	Men			
Cigarettes smoked *per day*, n	Age at cessation, years			
	25–34	35–44	45–54	55–64
1–9	4 (48)	3 (38)	3 (29)	2 (21)
10–19	7 (46)	7 (36)	6 (27)	5 (19)
>20	9 (45)	8 (35)	7 (26)	6 (18)
	**Women**			
Cigarettes smoked *per day*, n	Age at cessation, years			
	25–34	35–44	45–54	55–64
1–9	3 (52)	3 (43)	3 (33)	2 (24)
10–19	5 (51)	5 (41)	4 (32)	3 (23)
>20	6 (50)	6 (41)	5 (31)	4 (22)

### 3.2. Comparison with Previous Studies

The present results, which are the first from Southern Europe, show that the estimated life extension in FS from the Italian population is consistent with that observed in longitudinal studies conducted worldwide. Overall, male and female FS from Italian population, as well as those from the UK [4,5], US [6,7], and Japanese [10] population, gain the same life expectancy of never smokers (up to 10 years of life extension) when quitting before the 35 years of age, whilst even the oldest quitters obtain a not negligible increase in the number of years of life. In the study by Doll and Peto (more than 34,000 subjects followed since 1951 to 2001), male British doctors who quit at 30, 40, 50, or 60 years gain, respectively, about 10, 9, 6, or 3 years of life expectancy [4]. In the Million Women Study (1.3 million UK women enrolled in 1996–2001 and resurveyed about 3 and 8 years later), UK women who quit before 40 and 30 years of age avoid more than 90% and more than 97% of their excess mortality, respectively [5]. Based on data from CPS2 (a prospective study started in 1982 on a cohort of 1.2 million US adults over 30 years of age), life expectancy among US smokers who quit at age 35 exceeds that of continuing smokers by 6.9 to 8.5 years for men and 6.1 to 7.7 years for women; also those who quit at age 65 yields some benefits (1.4 to 2.0 years of life for men, and 2.7 to 3.7 for women) [6]. The more recent analyses of data from the U.S. National Health Interview Survey (113,752 women and 88,496 men, 25 years of age or older, interviewed between 1997 and 2004) shows that those smokers who quit at age 25–34, 35–44, or 45–54 gain about 10, 9, and 6 years of life, respectively, as compared to those who continue to smoke. In the Life Span Study (a cohort-study on 27,311 men and 40,662 women, followed from 1963 up to 2008), Japanese smokers who quit before age 35 avoid almost all of the excess risk among continuing smokers, while those who quit before age 45 avoid most of it [10].

When estimating mortality in relation to smoking, existing longitudinal studies do not distinguish for the number of smoked cig/day. The number of years of life gained with quitting smoking at various ages that is computed in the above quoted studies [4,6] is similar to that estimated in this study for smokers more than 20 cig/day. For example, Italian men smoking >20 cig/day who quit at 25–34, 35–44, 45–54, 55–64 years of age could gain 9, 8, 7, and 6 years of life expectancy, respectively. Corresponding figures from the study by Doll *et al.* [4] were 10, 9, 6, and 3, while Taylor *et al.* [6] found that men who quit smoking at 35, 45, 55, and 65 years of age could gain about 8, 7, 5, and 2 of life expectancy, respectively.

### 3.3. Gender Related Differences

We observed that the number of years of life gained with smoking cessation was higher in men than in women, particularly in those who smoked more than 10 cig/day and quit after age 35. This finding may be explained by the heavier smoking history of Italian men [18], which may have affected the magnitude of the RRs. Indeed, the pattern of smoking habit in Italy is characterized by a higher CS prevalence rate in men than in women in the last sixty years, a higher mean daily cigarettes consumption in men than in women, and a dramatic decrease of CS prevalence rates in men since 1957 (65%) to 1990 (38.3%) against a corresponding increase in women (6.2 to 25.9%) in the same period [13,18]. Indeed, the decreasing trend has continued throughout 2013: prevalence of male and female smokers is 26.2 and 15.3%, respectively [20].

New findings from prospective studies confirm the previous prediction that “If women smoke like men they will die like men” [21]. Measuring temporal trends in mortality across three time periods (1959–1965, 1982–1988, and 2000–2010), Thun *et al.* have shown that RRs for lung-cancer death among smokers were almost five times as high for men, as compared to women, in the 1959–1965 cohort, but in the 2000–2010 cohort the risks had equalized and had increased at 25 times as high for both men and women [8]. The women aged 25 or older, interviewed between 1997 and 2004 in the U.S. National Health Interview Survey, represent the first generation in the US in which those who smoked began early in life and smoked for decades; for these women Jha *et al.* found a tripled RR of death and a reduction in survival by at least a decade as for men [7]. In the 1920–1945 Japanese cohort (in which men and women started smoking before age 20, and smoked a similar number of cig/day), those who continued smoking had an overall mortality, *versus* never smokers, more than doubled in both sexes and life expectancy reduced by almost a decade (8 years for men, 10 years for women) [10].

### 3.4. Clinical Implications

Despite the decrease in smoking prevalence the proportion of annually quitters, however, was estimated to be very low in Italy: It was higher than 5% only in elderly persons and in women aged <30 years, while in adults aged 30–49 and 50–59 cessations were about 2% and 3–5%, respectively. In addition, most of quit probabilities stalled from 1986 to 2009 [22,23]. 

Apart nicotine dependence, psychological distress, personality traits and others [24,25,26] as potential factors affecting smoking cessation, it is recognized that the motivation to quit is a basic requisite for starting an attempt to stop smoking [27]. In a recent study on 3,075 former smokers from a representative sample of the Italian adult population, the second more prevalent motivation reported to stop smoking—after “a current health condition” (43.2%)—was “to avoid future health problems” (31.9%) [28].

Improving communication on smoking-related health problems may help to trigger the decision to quit smoking. Hazards that smokers have to face may be explained by using risk charts [16,29], while receiving spirometry results with pictorial feedback on “Lung age” instead of just raw data improves quit rates [30]. 

By using individual information (gender, age, smoked cig/day), the present study estimates the additional number of years that a single male or female smoker can live if he/she stops smoking at once. Then, the “Life gain” may be also computed as percentage increase of estimated years gained with smoking cessation over the years of life expectancy if continuing smoking. Such information could be used by physicians and health personnel to promote a quit attempt. A software (available at the website www.6elle.net) was developed to compute a personalized report on the additional number of life years and the percentage reduction of the risk of dying in the next 10 years for the main tobacco-related diseases (*i.e.*, lung cancer, acute myocardial infarction, Chronic Obstructive Pulmonary Disease, and stroke) that could be gained by stopping smoking. A randomized controlled study would be worthwhile in order to validate the efficacy of this report in improving quit rates in the clinical routine. 

### 3.5. Limits and Strengths

Following the approach of Woloshin *et al.* [16], when computing life tables specific for CS, NS, and FS, age and sex-specific death rates were computed by using death for all causes, but it is expected that mortality—and accordingly survival—for smoking-related causes of death is higher. For example, Thun *et al.* have shown that relative risks for death in CS were 25.61 in men and 22.35 in women for Chronic Obstructive Pulmonary Disease, while they were 2.80 in men and 2.76 in women for all causes combined [8].

The present approach in estimating life gains does not take into account confounders other than gender, cig/day, and age of quitting. However, when some confounders were considered in the prospective study by Doll *et al.* [4] (*i.e.*, alcohol consumption, body mass index, blood pressure), they did not greatly affect the absolute difference between the overall mortality rates of cigarette smokers and lifelong non-smokers. It should be noted that our results on life expectancy are of the same magnitude of those observed in that prospective study. Estimates of smoking related risk scarcely changed after adjusting for possible confounders also in other studies [5,7].

Our analyses did not control for duration of smoking, because this information was not available. However, since age is highly correlated with duration of smoking, by taking into account age at quitting smoking, duration of smoking has been at least in part considered.

We used Italian figures except for the RRs of death, which were extracted from CPS1 and CPS2. However, such estimates are consistent with those derived from Italian epidemiological studies [31]. In addition, RRs were adjusted accordingly with those from the more recent CPS2, and risks for men were further readjusted to agree with those estimated for a different European Country (Norway) [17]. 

To our knowledge, this is the first study that estimates the gain in life expectancy with quitting smoking in smokers living in the Mediterranean area. The consistency of our results with those from longitudinal cohort studies conducted worldwide confirms the strength of the statistical approach we have used, which, as a result, might be also applied to other populations.

Finally, an important novelty of our analysis is having stratified by the number of smoked cig/day the estimates of the years gained by quitting smoking. Our results show that the proportions of survival, as well as the gains in life expectancy, in addition to the age at cessation, were importantly affected by cigarettes consumption in both sexes.

## 4. Conclusions

Consistently with the few prospective studies conducted worldwide, the present study based on Italian figures confirms that quitting smoking increases life expectancy for men and women, regardless of age and number of cig/day smoked at the time of cessation. The estimates of the number of years of life that could be gained by quitting smoking, when computed specifically for a single smoker, could be used by physicians and health professionals to promote a quit attempt.
