# *Correction*: Carrozzi, L.; *et al.* Life Gain in Italian Smokers Who Quit. *Int. J. Environ. Res. Public Health* 2014, *11*, 2395–2406

**DOI:** 10.3390/ijerph110605970

**Published:** 2014-06-05

**Authors:** Laura Carrozzi, Franco Falcone, Giulia Carreras, Francesco Pistelli, Giuseppe Gorini, Andrea Martini, Giovanni Viegi

**Affiliations:** 1 Pulmonary Unit, CardioThoracic and Vascular Department, University Hospital of Pisa, via Paradisa 2, Cisanello, Pisa 56124, Italy; E-Mails: l.carrozzi@ao-pisa.toscana.it (L.C.); f.pistelli@ao-pisa.toscana.it (F.P.); 2 Unit of Pulmonary Environmental Epidemiology, Institute of Clinical Physiology, Italian National Research Council (IFC-CNR), via Trieste 41, Pisa 56126, Italy; E-Mail: viegig@ifc.cnr.it; 3 Italian Association of Hospital Pulmonologists (AIPO) Research, via Antonio Da Recanate 2, Milan 20124, Italy; E-Mail: franco.falcone@aiporicerche.it; 4 Unit of Environmental & Occupational Epidemiology, Cancer Prevention & Research Institute (ISPO), via delle Oblate 2, Florence 50139, Italy; E-Mails: g.gorini@ispo.toscana.it (G.G.); a.martini@ispo.toscana.it (A.M.); 5 Institute of Biomedicine and Molecular Immunology, Italian National Research Council (IBIM-CNR), via Ugo La Malfa 153, Palermo 90146, Italy

The authors wish to add the following amendments and corrections on their paper published in IJERPH [1].

1. Page 2399, Figure 3. Death rates for never smokers were computed using death rates for current and former smokers specific for each class of cigarettes smoked per day (1–9 cig./day, 10–19 cig./day, ≥20 cig./day). As a consequence, death rates for never smokers resulted cig./day-specific and not uniquely defined. To solve this problem we computed death rates for never smokers using overall prevalences and RR for current and former smokers (not cig./day-specific). Death rates for never smokers were however not used to compute the life gains. Due to this, replace:

**Figure 3:**
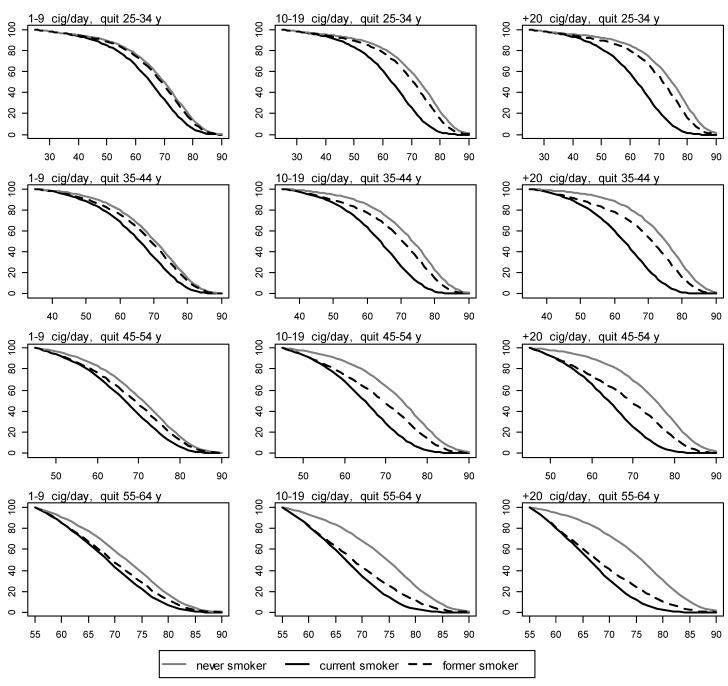
Survival for never, current, and former men smokers by number of cigarettes smoked per day and age of quitting. y axis: proportion of survival. x axis: age in years.

With:

**Figure 3:**
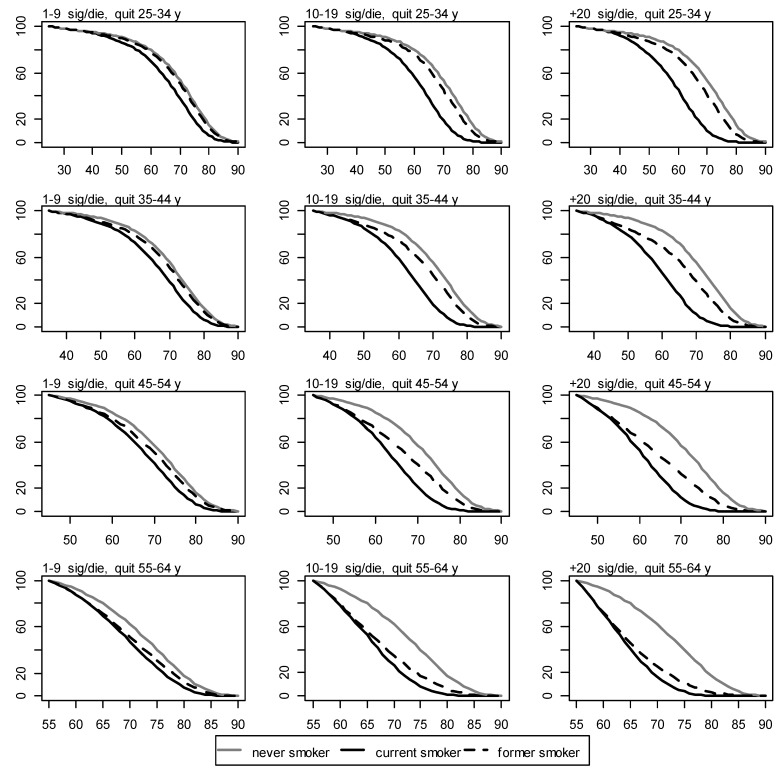
Survival for never, current, and former men smokers by number of cigarettes smoked per day and age of quitting. y axis: proportion of survival. x axis: age in years.
2. Page 2400, Figure 4. Due to the changes in Figure 3, replace:

**Figure 4:**
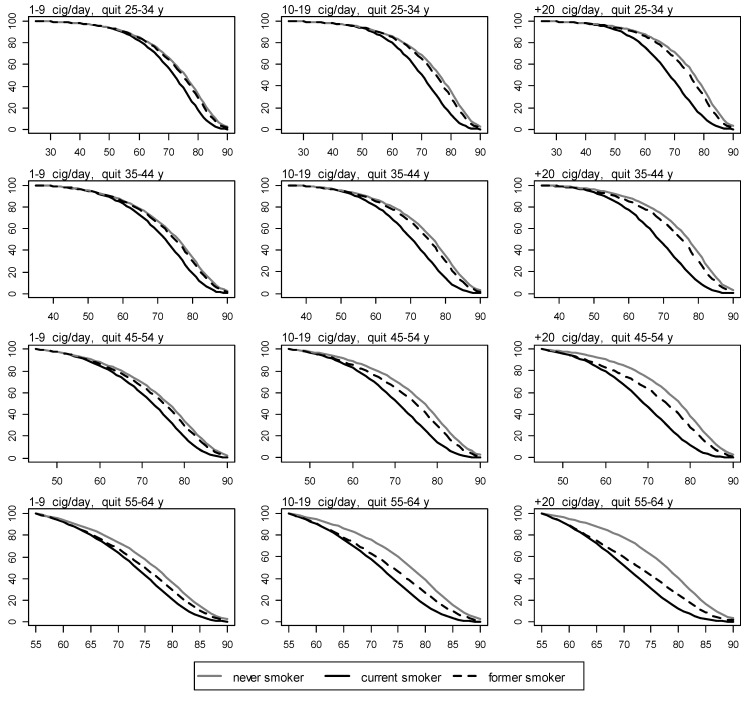
Survival for never, current, and former women smokers by number of cigarettes smoked per day and age of quitting. y axis: proportion of survival. x axis: age in years.

With:

**Figure 4:**
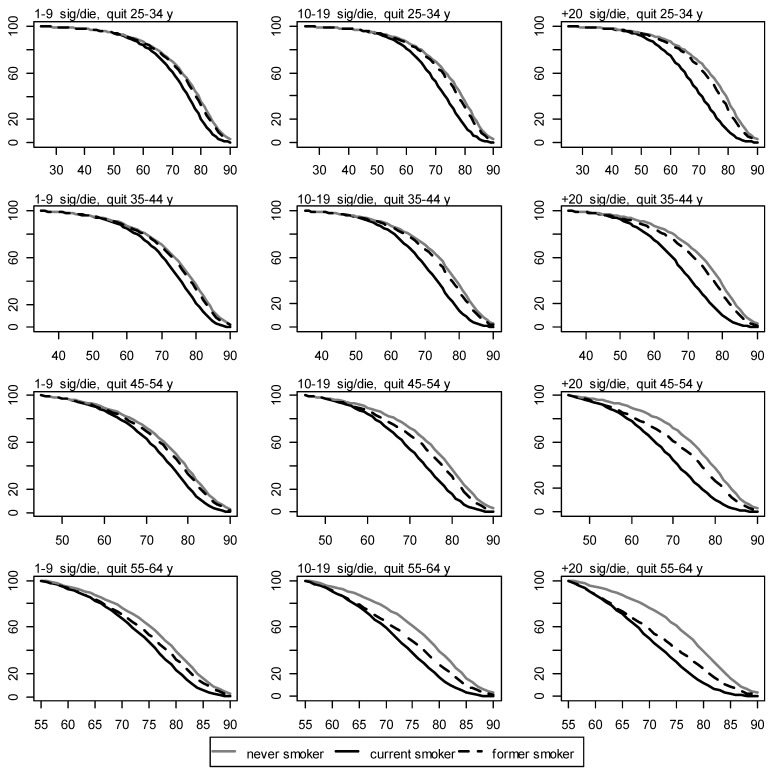
Survival for never, current, and former women smokers by number of cigarettes smoked per day and age of quitting. y axis: proportion of survival. x axis: age in years.

The authors would like to apologize for any inconvenience caused to the readers by these changes.
